# Traumatic Fetal Intracranial Hemorrhage Suggested by Point-of-Care Ultrasound

**DOI:** 10.5811/cpcem.2017.11.36214

**Published:** 2018-01-18

**Authors:** Nicole Chicoine Mooney, Lei Wu, Michael E. Vrablik

**Affiliations:** * University of Washington, Department of Emergency Medicine, Seattle, Washington; † University of Washington, Department of Radiology, Seattle, Washington

## Abstract

While the use of ultrasound to diagnose a fetal intracranial hemorrhage in utero is not a new concept, the emphasis of point-of-care ultrasound (POCUS) at the initial trauma presentation of the mother to evaluate for fetal injury is novel. A review of the literature failed to reveal a single case report wherein POCUS in the workup of a pregnant trauma patient led to the diagnosis of fetal intracranial hemorrhage. This is such a case.

## INTRODUCTION

Prenatal detection of intracranial hemorrhage is extremely rare,^1^ estimated as only one per 10,000 pregnancies.^2^ Even rarer is the detection of fetal intracranial hemorrhage as a result of maternal trauma. While this report highlights a rare case of fetal intracranial hemorrhage due to maternal trauma, it is most noteworthy for the role that point-of-care ultrasound (POCUS) played in the initial evaluation of the mother, which ultimately led to the discovery that the fetus had suffered an intracranial hemorrhage. No similar report can be found in the literature that describes the use of POCUS to evaluate a pregnant trauma patient at the initial presentation in the emergency department (ED) and ultimate discovery of fetal intracranial injury. This case also demonstrates the extensive and unique coordination required of the emergency medicine (EM), obstetrics, neurosurgery, medicine, surgery, and radiology services to diagnose and manage the injuries of both the patient and her fetus.

## CASE REPORT

The patient was a 46-year-old, gravida five, parity four (G5P4) at 24 weeks and five days (24w5d) gestation by last routine ultrasound, who presented to our Level 1 trauma ED after being a front-seat restrained (by a lap-shoulder belt) passenger in a high-speed rollover motor vehicle collision (MVC), with unknown loss of consciousness. The patient arrived hemodynamically stable but complaining of abdominal, pelvic, and back pain. She had no personal or family history of any coagulation disorder, was not being treated with any anticoagulant, and denied any use of illicit drugs.

Initial examination revealed a gravid woman in a cervical spine collar, on a backboard, and moving all extremities. Vital signs were pulse of 98 beats per minute; blood pressure of 108/59 millimeters of mercury; respiration of 24 breaths per minute; and oxygen saturation of 99% on room air. The secondary survey revealed two facial lacerations, thoracic (T) and lumbar (L) spinal tenderness, a gravid uterus, tender abdomen, abdominal ecchymosis consistent with a seat-belt sign, and reduced strength in the right lower extremity, but otherwise neurologically intact. The initial pelvic radiograph revealed a left sacral fracture. Cardiotocographic monitoring revealed a fetal heart rate ranging from 130s-170s, with periodic decelerations.

**Image 1:**
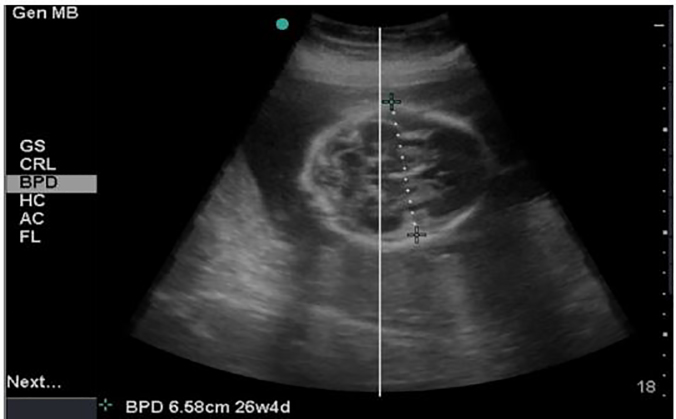
Emergent point-of-care transabdominal ultrasound demonstrated a biparietal diameter of 6.58 centimeters - consistent with a fetal gestation of 26 weeks 4 days. Based on the patient’s reported gestational age, the fetal gestation should have been 24 weeks 5 days. This discrepancy raised concern for fetal intracranial injury.

POCUS was performed using a SonoSite Micomaxx (FUJIFILM SonoSite, Inc.), curvilinear transducer with abdominal and obstetric settings. An emergency extended focused assessment with sonography for trauma (eFAST) and POCUS, were performed by an EM resident and an ultrasound fellowship-trained EM attending, to evaluate for maternal injury and fetal viability, size, position, multiparity, age, and placental abruption. The eFAST was negative. The POCUS revealed a viable single fetus, a biparietal diameter of 6.58 centimeters (cm), consistent with a gestation of 26w4d. Based on the patient’s stated gestational age, the fetal gestation should have been 24w5d. This discrepancy of almost two weeks was abnormal. The emergency physicians were concerned for fetal intracranial injury, prompting further fetal imaging and immediate consultation with obstetrics (Image 1)*.*

A formal obstetric ultrasound was subsequently performed, which revealed a biparietal diameter of 6.8 cm and head circumference of 24.5 cm, consistent with fetal gestation of 27w3d and 26w5d respectively, bilateral extra-axial fluid collections concerning for subdural hematomas, and no placental abruption. A subsequent ultrasound with color and Doppler of fetal middle cerebral artery was performed, which revealed normal bilateral Doppler.

**Image 2:**
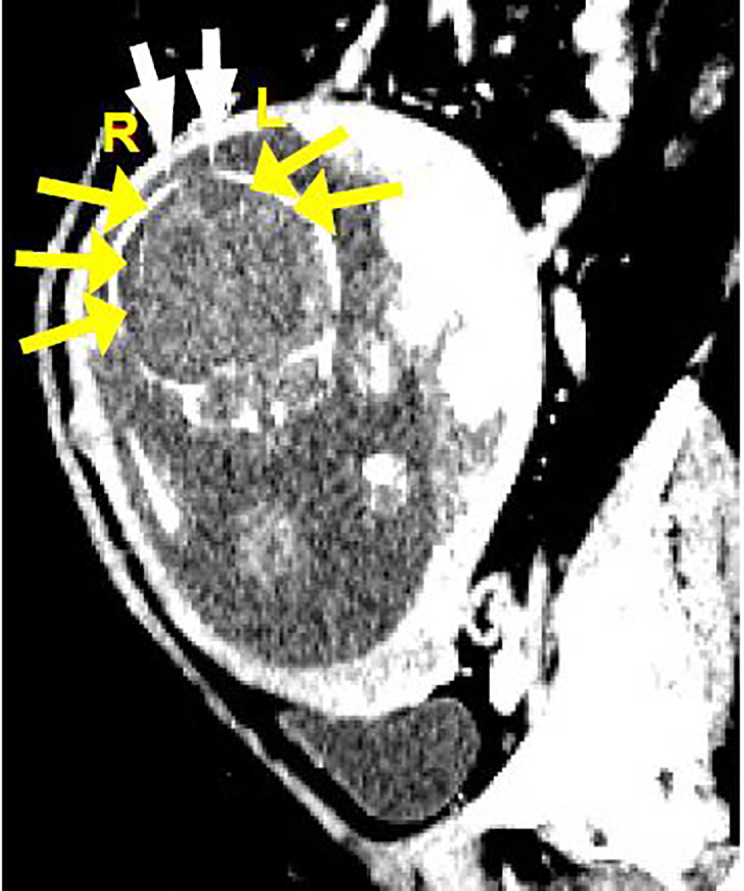
Computed tomography angiography abdomen and pelvis sagittal view revealed bilateral subdural hematomas (yellow arrows) and fetal skull diastasis (white arrows).

Based upon the mechanism of injury and physical examination, the patient underwent a computed tomography (CT) of the head and full spine, with CT angiography (CTA) of the abdomen and pelvis. The CT head revealed a left mandibular and right nasal arch fractures. The CT full spine was notable for a three-column fracture of the T12 vertebral body, bilateral T12 and L1 transverse process fractures, and an oblique fracture of L1 vertebral body. The CTA revealed 10^th^ and 11^th^ left posterior rib fractures, fetal subdural hematomas, and fetal skull diastasis (Image 2)*.*

Neurosurgery determined that the patient’s spinal fractures were unstable, requiring full T&L spine precautions and surgical intervention for decompression and stabilization. A joint decision was made by the EM, obstetrics, neurosurgery, surgery, and medicine services to admit the patient to the medicine intensive care unit for observation and spinal precautions, a planned Cesarean section (C-section) at a later date, provided the fetus remained stable, and maternal spinal surgery immediately after delivery. Simultaneous delivery of the fetus and spinal surgery for the patient was determined to be too risky to both the patient and fetus.

CPC-EM Capsule

What do we already know about this clinical entity?

Fetal intracranial hemorrhages are rare. No etiology is found in most cases. Fetal intracranial hemorrhages are usually detected at routine prenatal sonography.

What makes this presentation of disease reportable?

A point-of-care ultrasound (POCUS) used in evaluating a trauma patient led to the discovery that her fetus had suffered an intracranial bleed. No similar report can be found in the literature.

What is the major learning point?

Consider a POCUS in the evaluation of the gravid trauma patient. Any discrepancy in biparietal measurement with gestational age should raise concern for fetal intracranial injury.

How might this improve emergency medicine practice?

This case demonstrates the value in performing a POCUS when evaluating the gravid trauma patient. It can aid in the early detection of fetal intracranial injury, as well as maternal injury.

The patient’s spinal precautions included log roll procedures with turning and lying flat on an automatic tilting bed for the duration of her pregnancy. (The patient was unable to wear a thoracolumbosacral orthosis due to her gravid uterus.) At 33w6d, 64 days after the patient’s accident, while on a backboard for spinal precautions, the patient underwent a C-section, delivering a viable male fetus with encephalomalacia and microcephaly likely due to the bilateral subdural hematomas. Immediately post-delivery, the patient underwent spinal imaging, which revealed stable spinal fractures that no longer required surgery. The patient was eventually moved to a rehabilitation facility for intensive physical therapy. At time of discharge, the patient’s son suffered from encephalomalacia with microcephaly, mild extremity hypertonia, hyper-reflexia, and aspiration with feeding requiring a nasal-gastric tube.

## DISCUSSION

Causes of extremely rare fetal intracranial hemorrhages include maternal trauma, maternal and fetal coagulation disorders, twin-to-twin transfusion syndrome, maternal use of warfarin or cocaine, and maternal infection.^1^,^2^,^3^ No etiology is found in most cases.^1^, ^2^,^3^ Fetal intracranial hemorrhage in the circumstance of maternal trauma tends to be even rarer due to the fact that the cushion of the uterus and amniotic fluid are protective.^4^ When such fetal injuries exist, it is evidence of the magnitude of blunt force to the maternal abdomen.^4^

Fetal intracranial hemorrhage may be epidural, subdural, subarachnoid, intraventricular or intraparenchymal.^3^ Intraventricular hemorrhage is the most common type of perinatal and neonatal intracranial hemorrhage.^4^ Epidural and subdural hematomas are most often due to trauma.^1^ Most intraventricular hemorrhages are the result of thrombocytopenia, fetal coagulation disorders or hypoxia.^1^ The prognosis of fetal intracranial hemorrhages is poor, with approximately 40% of fetuses dying in utero or within the first month of life.^2^,^3^ Of those that survive, 50% have neurological dysfunction or delay.^1^, ^2^,^3^ Subdural hematomas have the best prognosis.^1^

Most fetal intracranial hemorrhages are detected at routine prenatal sonography. A 2003 case report describes a 20-year-old woman, at 28w gestation, involved in a MVC.^4^ An ED POCUS failed to show any fetal abnormalities.^4^ The patient was taken urgently to the operating room for diaphragmatic repair, wherein the fetus decompensated intraoperatively, a placental abruption was identified, and an emergency C-section was performed.^4^ A formal, postpartum ultrasound of the newborn’s head revealed bilateral subdural hematomas, a left frontal and occipitotemporal intracranial hemorrhages, later confirmed by CT.^4^ The failure of the ED POCUS to identify any fetal intracranial hemorrhage was likely due to limited technology and image quality at the time.

Fetal intracranial hemorrhage can be challenging to identify.^2^ Sonography is the diagnostic modality of choice.^3^ Magnetic resonance imaging (MRI) often follows to confirm the presence of an intracranial hemorrhage, as ultrasound has low sensitivity for small hemorrhages, and an MRI is helpful for estimation for time of and evolution of the bleeding.^3^ Sonographic evidence of a fetal intracranial hemorrhage depends upon the timing of the ultrasound in relation to the insult.^1^ If the bleed occurs within 24–48 hours of the ultrasound, the sonographer should see hyperechoic signals without posterior shadowing.^1^,^2^ After 48 hours, as blood reabsorption begins, the sonographer should see varying degrees of echo densities.^1^ Ultimately, the area of the bleed will become anechoic and a cyst may form.^1^,^2^,^3^

An MRI should be subsequently performed to confirm the extent and timing of the fetal intracranial hemorrhage and for further evaluation of the fetal brain. An MRI has higher sensitivity and specificity than sonography and carries no radiation risk. However, in the setting of significant maternal trauma, it is imperative that catastrophic injuries of the patient be identified emergently. CT imaging (as opposed to MRI) should be performed, despite the radiation risk, as it is readily available and offers better temporal and spatial resolution.

## CONCLUSION

While this case is a very rare event of a fetal intracranial hemorrhage in the setting of maternal trauma, it is novel in that it demonstrates the value of POCUS in an attempt to identify early fetal injury. It also emphasizes the extensive coordination required to diagnose and manage both the traumatic injuries of both the patient and the fetus.
